# On the Iterative Methods of Linearization, Decrease of Order and Dimension of the Karman-Type PDEs

**DOI:** 10.1155/2014/792829

**Published:** 2014-03-16

**Authors:** A. V. Krysko, J. Awrejcewicz, S. P. Pavlov, M. V. Zhigalov, V. A. Krysko

**Affiliations:** ^1^Engels Institute of Technology (Branch), Saratov State Technical University, Department of Higher Mathematics and Mechanics, Russian Federation, Ploschad Svobodi 17, Engels, Saratov 413100, Russia; ^2^Department of Automation and Biomechanics, Lodz University of Technology, and Department of Vehicles, Warsaw University of Technology, 84 Narbutta Street, 02-524 Warszawa, Poland; ^3^Saratov State Technical University, Department of Mathematics and Modeling, Russian Federation, Polytehnicheskaya 77, Saratov 410054, Russia

## Abstract

Iterative methods to achieve a suitable linearization as well as a decrease of the order and dimension of nonlinear partial differential equations of the eighth order into the biharmonic and Poisson-type differential equations with their simultaneous linearization are proposed in this work. Validity and reliability of the obtained results are discussed using computer programs developed by the authors.

## 1. Introduction

Mathematical models of continuous mechanical structures are described by nonlinear partial differential equations which may be solved analytically only in a few rare cases. However, a direct application of the numerical methods is associated also with big difficulties regarding a high order of both dimension and differential operator, as well as nonlinearity of the PDEs studied.

This is why it is tempting to develop approaches that offer a reduction of the input differential equations. The mentioned methods can be divided into three groups: (1) linearization; (2) order decrease of the PDEs; (3) order decrease of a differential operator.

The so far existing methods of solutions of nonlinear problems, depending on the introduced linearization level, can be divided into two groups. The first one deals with the linearization of PDEs, whereas the second one is dedicated to the linearization of algebraic equations obtained through the discretization procedures applied to the input PDEs. Below, we consider the methods associated with the first group. This group contains the Newton and Newton-Kantorovich methods [1].

One of the linearization methods is the method of quasilinearization, widely illustrated in monograph [2]. It presents a further development of Newton's method, and it generalizes the method proposed by Kantorovich.

On the other hand, there is a seminal approach known as the Agmon-Douglis-Nirenberg (ADN) theory for elliptic PDEs still attracting a large number of imitators [3, 4]. In particular, the abstract least squares theory is developed satisfying the ADN elliptic theory assumptions [5–7].

Furthermore, in the case of corners in plane domains the ADN system exhibits singularities, which imply a need for construction of singular exponents and angular functions [8]. Our approach does not have this disadvantage and it is simple in direct applications to the real world systems.

The so far briefly addressed approaches linearize the input problem; that is, they reduce it to the solution of linear problems. However, there is one more important question to be solved, that is, a reduction of the space dimension of the initial problem.

One of the methods to solve the stated problem is focused on averaging (integration) along such a coordinate on which the object dimension is lesser in comparison to the two remaining coordinates. On the other hand, it is well known that mathematical problems related to the theory of material strength can be formulated as variational problems, that is, the problems of finding extrema of a certain functional. Variational statements create a foundation for the construction of direct difference and variational methods, as it is widely described in monograph [9].

We mention only a few works [10–12] devoted to the third group, that is, aiming at a decrease of the PDE order.

Note that the so far presented state of the art of the proposed and applied methods allows us to solve each of the mentioned problems separately: either a decrease of the system order or its linearization. However, we show how all these problems can be solved simultaneously.

Our paper is focused mainly on the method of dimension decrease and linearization of the Karman-type PDEs. However, the presented approach can be successfully applied to other nonlinear PDEs. In particular, in the modified version two variants of the proposed method are presented:

- the first iterative method consists of the reduction of the eighth order linear PDEs to a successive solution of linear PDEs of the fourth order biharmonic equations; that is, the system dimension is reduced twice with simultaneous linearization of the problem;
- the applied second iterative procedure includes a further order decrease of the earlier obtained (first iterative method) linear system of biharmonic PDEs of the fourth order to the successive solution to the system of the second order Poisson-type equations.

In other words, the application of these two iterative procedures implies a fourfold reduction of the PDEs order with the linearization procedure carried out simultaneously.

The proposed iterative procedures regarding the nonlinear PDEs order decrease and linearization can also be applied to PDEs with a curvilinear boundary. The application of FDM (finite difference method) to solve biharmonic equations and PDEs of the Poisson-type requires a solution to the so-called Sapondzhyan-Babuška problem. The paradox of Sapondzhyan-Babuška (see [13–15]) was discovered when studying the asymptotic behavior of solutions to an elasticity system in a thin polygonal plate (inscribed in the plate with smooth boundary) as the length of the side of the polygon tends to zero and the number of sides goes to infinity. In Section 2 of our work we prove the proposed iterative procedure to remove this paradox (this problem concerns smoothness of the curvilinear boundary).

In Section 3 of our work the reliability and validity of the method of variational iterative procedure to solve PDEs described by positively defined operators are illustrated and discussed. Namely, the convergence of the method of variational iterations generalizes the Kantorovich-Vlasov method [16] aimed at the reduction of PDEs to ODEs. On the other hand, as it was pointed out by Vorovich [17], the Kantorovich-Vlasov method generalizes the Galerkin method. It should be emphasized that the choice of approximating functions referring either to two variables in the Galerkin method or to one variable in the Kantorovich-Vlasov method cannot be applied in the method of variational iterations. The system of functions being sought is provided by a solution of the PDEs with regard to two variables assuming that we deal with the 2D problem. Furthermore, the proposed approach can be applied to 3D elliptic equations.

Section 6 of the paper deals with a comparison of the solutions to the Karman equations obtained via our proposed iterative procedures with those offered by FEM and FDM, as well as with experimental results. Good coincidence of the results is achieved.

## 2. Mathematical Model of a Flexible Karman-Type Plate (Hypotheses, Differential Equations, and Boundary Conditions)

**Figure 1:**
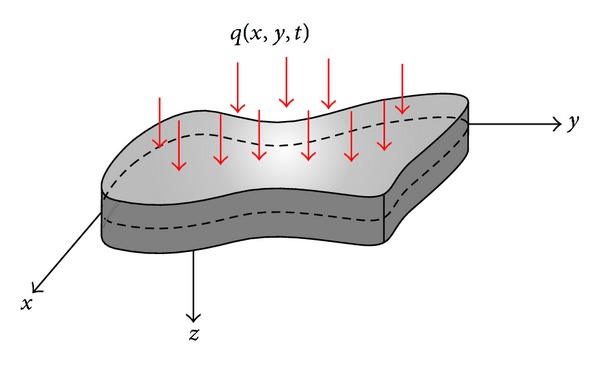
A studied plate.

The objects of our investigation are plates of different shapes (in particular, rectangular ones), representing a closed 3D part of space *R* ^3^ (Figure 1). The following hypotheses are introduced: (i) plate material is elastic and isotropic; (ii) the following Karman relations between deformations and displacements are introduced:

(1) $$\begin{array}{c} \varepsilon_{xx}=\frac{\partial u}{\partial x}+\frac{1}{2}{\left(\frac{\partial w}{\partial x}\right)}^{2},\;\varepsilon_{xy}=\frac{\partial u}{\partial y}+\frac{\partial v}{\partial x}+\frac{\partial w}{\partial x}\frac{\partial w}{\partial y},\;\\ \left(u,v,w\right),\left(x,y\right).\; \end{array}$$

Equations governing the deflection *w*(*x*, *y*, *t*) and stress function *F*(*x*, *y*) have the following form [15]:

(2) $$\begin{array}{c} \Delta^{2}w-L\left(w,F\right)-q=0,\\ \Delta^{2}F+\frac{1}{2}L\left(w,w\right)=0. \end{array}$$

The following operators are introduced:

(3) Δ2(·)=1λ2∂4(·)∂x4+λ2∂4(·)∂y4+2∂4(·)∂x2∂y2,L(·,·)= ∂2(·)∂x2∂2(·)∂y2−2∂2(·)∂x ∂y∂2(·)∂x ∂y+∂2(·)∂x2∂2(·)∂y2.

Here and further on the following nondimensional quantities are introduced: $w=h\overset{-}{w}$; $F=Eh^{2}\bar{F}$; $t=t_{0}\bar{t}$; $\varepsilon =\bar{\varepsilon }/\tau$; $x=a\bar{x}$; $y=b\overset{-}{y}$; $q=\bar{q}(12(1-\nu^{2})Eh^{4}/a^{2}b^{2})$; $\tau =(ab/h)\sqrt{\rho /Eg}$; *λ* = *a*/*b*, where *a*, *b* are the maximal plate dimensions regarding *x* and *y*, respectively; *h* is thickness; *g* is acceleration due to gravity; *ρ* = *γh*; *γ* is specific gravity of volume plate material; *ν* is Poisson's coefficient; *E* is the Young modulus; *w*, *F* are deflection and stress functions, respectively.

Let us add boundary conditions of the support on flexible nonstretched (noncompressed) ribs to the system of plates [18, 19]:

(4) $$\begin{array}{c} {w|}_{\Gamma }={\frac{\partial^{2}w}{\partial n^{2}}|}_{\Gamma }={F|}_{\Gamma }={\frac{\partial^{2}F}{\partial n^{2}}|}_{\Gamma }=0, \end{array}$$

where Γ stands for the space boundary occupied by the plate. The following initial conditions are attached to (2):

(5) $$\begin{array}{c} {w|}_{t=0}=w_{0},\;\;{\dot{w}|}_{t=0}={\dot{w}}_{0}. \end{array}$$

System of (2) is composed of nonlinear PDEs of the eighth order. Finding a reliable solution to this problem is still a serious problem in spite of achievements of the numerical methods. It should be emphasized that the solution to the mentioned problem was found earlier via either FDM (finite difference method) or FEM (finite element method), or by the Bubnov-Galerkin method. Below, we propose a novel method of order reduction and linearization of PDEs (2).

## 3. Methods of Order Decrease and Linearization of the Karman Equation

There are two ways for construction of the fundamental iterative procedure to solve system (2): (i) system reduction to a successive solution to the Germain-Lagrange type equations, in this case the system order is decreased twice; (ii) system reduction to a Poisson-type equation (in this case the system order is reduced four times). In both mentioned cases the linearization procedure of the input PDEs is carried out.

### 3.1. Iterative Linearization Procedure and Reduction of the Karman Equation into Germain-Lagrange Equations

We keep the biharmonic operator in each of (2), and we shift nonlinear terms into their right-hand sides. Assuming that functions on the right-hand sides are computed with respect to their previous step and that the equations are solved successively, the following iterative procedure is proposed:

(6) $$\begin{array}{c} \Delta^{2}w^{\left(k\right)}=L\left(w^{\left(k-1\right)},F^{\left(k-1\right)}\right)+q,\\ \Delta^{2}F^{\left(k\right)}=-\frac{1}{2}L\left(w^{\left(k\right)},w^{\left(k\right)}\right),\;\left\{x,y\right\}\in \Omega . \end{array}$$

On the first step of the iterative procedure the following biharmonic equation for a given load *q*(*x*, *y*) is solved:

(7) $$\begin{array}{c} \Delta^{2}w^{\left(1\right)}\left(x,y\right)=q\left(x,y\right). \end{array}$$

The value of *w* ^(1)^(*x*, *y*) is substituted into the right-hand side of equation system (6), and as a result a biharmonic equation for *F* ^(1)^(*x*, *y*) with the known right-hand side is obtained. The value of the stress function found so far is substituted to the first system equation. The process of finding solutions is continued to achieve the required accuracy.

Let us note that as a result of the application of the iterative procedure, the Germain-Lagrange type system of equations are obtained.

Let us prove convergence of the constructed iterative procedure. Let *H* ^2^(*Ω*) refer to a Sobolev space of functions *ξ* = {*w*, *F*} such that

(8) $$\begin{array}{c} \xi \in L^{2}\left(\Omega \right),\;\;\frac{\partial \xi }{\partial x_{i}}\in L^{2}\left(\Omega \right),\\ \frac{\partial^{2}\xi }{\partial x_{i}\;\partial x_{j}}\in L^{2}\left(\Omega \right);\;i,j=1,2, \end{array}$$

where *L* ^2^(*Ω*) denotes the space of functions being summed with a square in *Ω*.

Let *H*_0_^2^(*Ω*) denote the closure of functions from *D*(*Ω*) (space of functions of class *C* ^*∞*^ in *Ω*, having compact carrier in *Ω*) in norm *H* ^2^(*Ω*):

(9) $$\begin{array}{c} H_{0}^{2}\left(\Omega \right)={\bar{D(\Omega )}}^{H^{2}(\Omega )}=\left\{\xi \in H^{2}\left(\Omega \right)\;|\;{\xi |}_{\Gamma }={\frac{\partial \xi }{\partial n}|}_{\Gamma }=0\right\}. \end{array}$$

Since space *Ω* is bounded and its boundary Γ is efficiently regular, then map *ξ* → ||Δ*ξ*||_0,*Ω*_ defines the norm in *H*_0_^2^(*Ω*) being equivalent to the norm generated by spaces *H* ^2^(*Ω*).

Assume that *q* ∈ *H* ^−2^(*Ω*) (*H* ^−2^(*Ω*) denotes a conjugation to *H* ^2^(*Ω*)). It is known [17] that in this case problems (2) and (4) have a solution (it may happen that it shall be nonunique).

*Novel Variation Formulation of the Problem*. Let us denote by (·, ·) a scalar product in *L* ^2^(*Ω*): (*ξ*, *η*) = ∫_*Ω*_ *ξη* *dΩ*, and by *β*(*w*, *F*, *μ*) a three linear form defined on (*H*_0_^2^(*Ω*))^3^:

(10) $$\begin{array}{c} \beta \left(w,F,\mu \right)=\left(\Delta F,\Delta \mu \right)+\frac{1}{2}\left(L\left(w,w\right),\mu \right). \end{array}$$

Let us define the set

(11) $$\begin{array}{c} M=\left\{w,F\in H_{0}^{2}\left(\Omega \right)\;|\;\forall \mu \in H_{0}^{2}\left(\Omega \right),\beta \left(w,F,\mu \right)=0\right\}, \end{array}$$

and square function *J*(*w*, *F*) : *M* → *R*

(12) $$\begin{array}{c} J\left(w,F\right)=\frac{1}{2}{||\Delta w||}_{0,\Omega }^{2}+\frac{1}{2}{||\Delta F||}_{0,\Omega }^{2}-\left(q,w\right). \end{array}$$

**Theorem 1**

The problem of minimizing (12) on set (11) has, at least, one solution.

**Proof**

Let {*w*_*n*_, *F*_*n*_} ∈ *M* be the minimizing series; that is, we have

(13) $$\begin{array}{c} J\left(w_{n},F_{n}\right)⟶\underset{\left\{w,F\right\}\in M}{\inf}J\left(w,F\right), \end{array}$$

which exists, since *J* is a square functional.

For arbitrary *w*, *F* ∈ *H*_0_^2^(*Ω*) the following inequality holds:

(14) $$\begin{array}{c} J\left(w,F\right)\geq c_{1}{||w||}_{2,\Omega }^{2}+c_{2}{||F||}_{2,\Omega }^{2}-c_{3}\cdot {||w||}_{2,\Omega }, \end{array}$$

where ||·||_2,*Ω*_ denotes the norm in *H* ^2^(*Ω*) and *c*_*i*_ are certain positive constants. Then, (13) yields *c*_1_||*w*_*n*_||_2,*Ω*_^2^ + *c*_2_||*F*_*n*_||_2,*Ω*_^2^ − *c*_3_||*w*_*n*_||_2,*Ω*_ ≤ *J*(*w*_*n*_, *F*_*n*_) ≤ *J*(*w*_0_, *F*_0_) = *A*, where *w*_0_, *F*_0_ are the arbitrary functions (initial approximation).

Then, the following estimation holds:

(15) $$\begin{array}{c} c_{1}\;{\left({||w_{n}||}_{2,\Omega }^{2}-\frac{c_{3}}{2c_{1}}\right)}^{2}+c_{2}\;{||F_{n}||}_{2,\Omega }^{2}\leq A+\frac{c_{3}^{2}}{4c_{1}},\\ {||w_{n}||}_{2,\Omega }\leq c_{4},\;\;{||F_{n}||}_{2,\Omega }\leq c_{5}. \end{array}$$

Therefore, series {*w*_*n*_, *F*_*n*_} is bounded in (*H*_0_^2^(*Ω*))^2^. Consequently, one may choose a series {*w*_*k*_, *F*_*k*_} that $w_{k}\to \tilde{w}$, $F_{k}\to \tilde{F}$ is weak in *H*_0_^2^(*Ω*). Since *H*_0_^2^(*Ω*) → *L* ^2^(*Ω*) is compact, then $w_{k}\to \tilde{w}$, $F_{k}\to \tilde{F}$ is strong in *L* ^2^(*Ω*).

We show that interval $\left\{\tilde{w},\tilde{F}\right\}$ of the minimized series belongs to *M*; that is, $\beta \left(\tilde{w},\tilde{F},\mu \right)=0$, for all *μ* ∈ *H*_0_^2^(*Ω*).

Since (*L*(*w*_*k*_, *w*_*k*_), *μ*) = (*L*(*w*_*k*_, *μ*), *w*_*k*_) ∀ *μ* ∈ *H*_0_^2^(*Ω*), $L\left(w_{k},\mu \right)\to L\left(\tilde{w},\mu \right)$ is weak in *H*_0_^2^(*Ω*), and $w_{k}\to \tilde{w}$ is strong in *L* ^2^(*Ω*), we get $\left(L\left(w_{k},w_{k}\right),\mu \right)=\left(L\left(\tilde{w},\tilde{w}\right),\mu \right)$ and consequently, $\beta \left(\tilde{w},\tilde{F},\mu \right)=0$ for all *μ* ∈ *H*_0_^2^(*Ω*). This means that

(16) $$\begin{array}{c} \left\{\tilde{w},\tilde{F}\right\}\in M. \end{array}$$

However, *J*(*w*, *F*) is half-continuous from below in weak topology on (*H* ^2^(*Ω*))^2^, and therefore the following inequality holds: $\lim_{\bar{k\to \infty }}J\left(w_{k},F_{k}\right)\geq J\left(\tilde{w},\tilde{F}\right)$.

Then (13) and (16) imply that $J\left(\tilde{w},\tilde{F}\right)\leq \inf_{(w,F)\;\in M}\;J\left(w,F\right)$. Therefore, the following equation holds: $J\left(\tilde{w},\tilde{F}\right)=\inf_{(w,F)\;\in M}\;J\left(w,F\right)$, which means that $\left\{\tilde{w},\tilde{F}\right\}\in M$ is a solution to the minimization problem.

Let us explain how points of the minimum of functional (12) are linked with solutions to problems (6) and (4). For this purpose a notation of weak solution shall be introduced.

A weak solution to problems (6) and (4) is defined by the pair of functions {*w*, *F*} ∈ *M*, satisfying the following:

(17) $$\begin{array}{c} \left(\Delta w,\Delta \mu \right)-\left(L\left(w,F\right),\mu \right)=\left(q,\mu \right)\;\forall \mu \in H_{0}^{2}\left(\Omega \right). \end{array}$$

**Theorem 2**

Points of the functional minimum (12) are weak solutions to problems (6) and (4).

**Proof**

Let {*w*, *F*} ∈ *M* be one of the functional (12) minimum points. Let us take *η* = *w* + *t* *δwδw* ∈ *H*_0_^2^(*Ω*) and let us choose *ξ* = *F* + *δFδF* ∈ *H*_0_^2^(*Ω*) such that {*η*, *ξ*} ∈ *M*, that is, in the way that *β*(*w*, *F*, *μ*) = 0   for all *μ* ∈ *H*_0_^2^(*Ω*). Then *J*(*w*, *F*) ≤ *J*(*η*, *ξ*). This yields

(18) t(Δw,Δδw)+(ΔF,ΔδF)−t(q,δw)+t22||Δδw||2,Ω2  +12||ΔδF||2,Ω2≥0, ∀t∈R,  δw∈H02(Ω),

and by taking *μ* = *F*, condition *β*(*η*, *ξ*, *μ*) = 0 yields

(19) $$\begin{array}{c} \left(\Delta F,\Delta \delta F\right)=-t\left(L\left(w,\delta w\right),F\right)-\frac{t^{2}}{2}\left(L\left(\delta w,\delta w\right),F\right). \end{array}$$

Substituting (19) into (18), dividing the obtained expression by *t* and going to the limit for *t* → 0, the following inequality is obtained:

(20) $$\begin{array}{c} \left(\Delta w,\Delta \delta w\right)-\left(L\left(w,F\right),\delta w\right)\geq \left(q,\mu \right). \end{array}$$

Substituting *δw* by −*δw* in (20), one obtains equality, that is, (17).

Let us use the following notation (Φ(*w*, *F*), *μ*) = *a*_1_(Δ*w*, Δ*μ*) − (*L*(*w*, *F*), *μ*) − (*q*, *μ*).

Equation (17) can be given in the following form:

(21) $$\begin{array}{c} \left(\Phi \left(w,F\right),\mu \right)=0, \end{array}$$

and it is clear that Φ(*w*, *F*) ∈ *H* ^−2^(*Ω*).

Therefore, each point of the minimum of functional (17) on *M* satisfies (21), and hence it is a weak solution to problems (6) and (4).

Therefore, it has been shown that finding a solution to problems (6) and (4) is equivalent to finding a solution to the problem of minimization (13) with the occurrence of constraints {*w*, *F*} ∈ *M*. The reduced problem can be solved by various methods to find a minimum taking into account the mentioned constarints. Once a solution to the problem of finding an extreme is chosen, various algorithms to solve problems (6) and (4) can be applied.

Below, we focus on the method of gradient projection with a restoring constraint [18], which for linear constraints allows for essential simplification of finding a solution to the stated problem.

Let us construct an iteration process of minimizing *J*(*w*, *F*) on *M* using the following scheme:

(a) element *w*_0_ ∈ *H*_0_^2^(*Ω*) is taken arbitrarily;
(b) after computation of *w*_*n*_, *F*_*n*_ ∈ *H*_0_^2^(*Ω*) and *w*_*n*+1_ ∈ *H*_0_^2^(*Ω*) is defined successively by solutions to the following problems:
  (22) $$\begin{array}{c} \beta \left(w_{n},F_{n},\mu \right)=0,\;F_{n}\in H_{0}^{2}\left(\Omega \right)\;\forall \mu \in H_{0}^{2}\left(\Omega \right), \end{array}$$
  (23) (Δwn+1,Δμ)=(Δwn,Δμ)−ρn(Φ(wn,Fn),μ)∀μ∈H02(Ω);
(c) coefficient *ρ*_*n*_ on step (b) is defined by the condition
  (24) J(wn+1,Fn+1)−J(wn,Fn)  ≤ε(Φ(wn,Fn),wn+1−wn), 0<ε<1,

where *ε* stands for a parameter of the method.

**Theorem 3**

For the iteration process (22) to (25) (Φ(*w*_*n*_, *F*_*n*_), *μ*) → 0 for *n* → 0 an arbitrary initial point {*w*_0_, *F*_0_} ∈ *M*, obtained through this procedure series {*w*_*n*_, *F*_*n*_} includes a subseries convergent to the weak solution to the problem ((6) and (4)).

**Proof**

A possibility of constructing the series {*w*_*n*_, *F*_*n*_} is yielded by an observation that for all *ρ*_*n*_ *w*_*n*+1_ ∈ *H*_0_^2^(*Ω*) and, consequently, *L*(*w*_*n*+1_, *w*_*n*+1_) ∈ *H* ^−2^(*Ω*), ∇_*k*_^2^ *w*_*n*+1_ ∈ *H* ^−2^(*Ω*) [19]. It means that the coupling equation *β*(*w*_*n*+1_, *F*_*n*+1_, *μ*) = 0 is solvable. Consider the following difference:

(25) ΔJn=J(wn+1,Fn+1)−J(wn,Fn)=12(Δ(wn+1−wn),Δ(wn+1+wn)) + 12(Δ(Fn+1−Fn),Δ(Fn+1+Fn)) −(q,wn+1−wn).

Owing to {*w*_*n*_, *F*_*n*_} ∈ *M*, {*w*_*n*+1_, *F*_*n*+1_} ∈ *M*, (25) gives

(26) $$\begin{array}{c} \Delta J_{n}^{}=\left(\Phi \left(w_{n},F_{n}\right),\delta w\right)+\frac{1}{2}{||\Delta \delta w||}_{0,\Omega }^{2}+\frac{1}{2}{||\Delta \delta F||}_{0,\Omega }^{2}, \end{array}$$

where *δw* = *w*_*n*+1_ − *w*_*n*_, *δF* = *F*_*n*+1_ − *F*_*n*_. Taking (23) into account, one observes that *δw* serves as a general solution to the boundary value problem:

(27) $$\begin{array}{c} \Delta^{2}\delta w=-\rho_{n}\Phi \left(w_{n},F_{n}\right),\;\delta w\in H_{0}^{2}\left(\Omega \right). \end{array}$$

Further on it means that

(28) $$\begin{array}{c} \delta w=-\rho_{n}G\left[\Phi \left(w_{n},F_{n}\right)\right], \end{array}$$

where *G*[•] : *H* ^−2^(*Ω*) → *H*_0_^2^(*Ω*) stands for the linear bounded operator being inversed to operator Δ^2^(•). Therefore

(29) ΔJn=−ρn(Φ(wn,Fn),G[Φ(wn,Fn)]) +12||Δδw||0,Ω2+12||ΔδF||0,Ω2.

Let us proceed to the second order terms. Taking in (11) *μ* = *δw* and applying (28), one gets

(30) ||Δδw||0,Ω2=−ρn(Φ(wn,Fn),δw)=ρn2(Φ(wn,Fn),G[Φ(wn,Fn)]).

Let us estimate the last term in (29). Since {*w*_*n*_, *F*_*n*_} ∈ *M* and {*w*_*n*+1_, *F*_*n*+1_} ∈ *M*, then for *δF* the following equation should be satisfied:

(31) (ΔδF,Δμ)+(L(wn,δw),μ)+(∇k2δw,μ)  +12(L(δw,δw),μ)=0  δF∈H02(Ω), ∀μ∈H02(Ω).

This, in particular, yields

(32) ||ΔδF||0,Ω≤c7(||L(wn,δw)||L1(Ω)+||L(δw,δw)||L1(Ω)+||∇k2δw||L1(Ω)).

However, *w*_*n*_ belongs to the bounded set in *H*_0_^2^(*Ω*) for arbitrary *n*. It implies that ||Δ*δF*||_0,*Ω*_ ≤ *c*_8_||Δ*δw*||_0,*Ω*_^2^ or equivalently

(33) $$\begin{array}{c} {||\Delta \delta F||}_{0,\Omega }^{2}\leq c_{9}\rho_{n}^{4}{\left(\Phi \left(w_{n},F_{n}\right),G\left[\Phi \left(w_{n},F_{n}\right)\right]\right)}^{2}. \end{array}$$

Substituting (30), (33) into (29), and taking into account both positive defiantness (in the sense of (Φ(*w*_*n*_, *F*_*n*_), *G*[Φ(*w*_*n*_, *F*_*n*_)]) ≥ *α*||Φ(*w*_*n*_,*F*_*n*_)||^2^) and the constraints of operator *G*[•], one gets

(34) ΔJn≤−ρnc10||Φ(wn,Fn)||2 ×(−1+ρn2+c1ρn32||Φ(wn,Fn)||2).

The latter estimation shows that the values *ρ*_*n*_ ≠ 0 are responsible for the satisfaction of inequality (24). For this purpose *ρ*_*n*_ should be chosen in the following way:

(35) $$\begin{array}{c} \frac{\rho_{n}}{2}+c_{1}\frac{\rho_{n}^{3}}{2}{||\Phi \left(w_{n},F_{n}\right)||}^{2}\leq 1-\varepsilon . \end{array}$$

It can always be done, since 0 < *ε* < 1.

Taking *ρ*_*n*_ in accordance with the algorithm applied so far, the following estimations are obtained on each step:

(36) $$\begin{array}{c} \Delta J_{n}\leq -\rho_{n}\varepsilon {||\Phi \left(w_{n},F_{n}\right)||}^{2}, \end{array}$$

which means that for the arbitrarily taken *n* we have *J*_*n*+1_ − *J*_*n*_ ≤ 0. Since functional *J* is bounded from below, the last inequality yields for *n* → *∞*  Δ*J*_*n*_ → 0. Besides, (36) gives

(37) $$\begin{array}{c} {||\Phi \left(w_{n},F_{n}\right)||}^{2}\leq \frac{-\Delta J_{n}}{\varepsilon \rho_{n}}. \end{array}$$

Let us emphasize that the so far introduced algorithm of the choice of *ρ*_*n*_ guarantees that for arbitrary *n* we have *ρ*_*n*_ ≥ *ρ*_0_ > 0. In fact, because Δ*J*_*n*_ ≤ 0, then

(38) $$\begin{array}{c} J\left(w_{n},F_{n}\right)\leq J\left(w_{0},F_{0}\right)=A. \end{array}$$

Owing to (38), norms ||*w*_*n*_||_2,*Ω*_, ||*F*_*n*_||_2,*Ω*_ are bounded. Therefore, also the norm ||Φ(*w*_*n*_, *F*_*n*_)|| is bounded. In addition, taking (37) into account, we have ||Φ(*w*_*n*_, *F*_*n*_)|| → 0 for *n* → *∞*, and consequently, also (Φ(*w*_*n*_, *F*_*n*_), *μ*) → 0 for *n* → *∞*  for all *μ* ∈ *H*_0_^2^(*Ω*). The occurrence of a convergent subseries follows now from a bound of norms ||*w*_*n*_||_2,*Ω*_, ||*F*_*n*_||_2,*Ω*_ (see proof of Theorem 1).

We have shown in the above the convergence of the reduction procedure of system (2) to the successive solution to the biharmonic Germain-Lagrange type equation. The applied procedure linearizes and decreases the order of the input equations. We have proposed a further development of this approach on the basis of reduction of the biharmonic equation to that of the Poisson-type. The latter approach allows us to decrease four times the order of system (2).

### 3.2. Iterative Procedure of Reduction of the Germain-Lagrange Equations Type to the Poisson Equations Type

The following original iterative procedure is proposed.

We consider a biharmonic equation given in the bounded convex space *Ω* ∈ *R* ^2^:

(39) $$\begin{array}{c} \Delta^{2}w\left(x,y\right)=g\left(x,y\right). \end{array}$$

On the space boundary the following boundary conditions are given:

(40) $$\begin{array}{c} {w|}_{\Gamma }=0,\;\;\Delta w-\chi {\frac{\partial w}{\partial n}|}_{\Gamma }=0, \end{array}$$

where *χ* denotes the curvature of boundary Γ.

Let us introduce the following new function *M*_*w*_ = Δ*w*. Substituting this function into (39) the following system of two Poisson-type equations is obtained:

(41) $$\begin{array}{c} \Delta M_{w}\left(x,y\right)=g\left(x,y\right),\\ \Delta w\left(x,y\right)=M_{w}\left(x,y\right),\;x,y\in \Omega . \end{array}$$

Boundary conditions have the following form:

(42) $$\begin{array}{c} {w(x,y)|}_{x,y\in \Gamma }=0,\;\;{M_{w}(x,y)|}_{x,y\in \Gamma }=0. \end{array}$$

Therefore, a solution of the biharmonic equation is divided into a solution of two Poisson-type equations. Below, we prove convergence of the proposed procedure.

Let us define the following set for the function *ξ* = {*w*, *M*_*w*_}:

(43) $$\begin{array}{c} E=\left\{\xi \in \rho^{\infty }\left(\Omega \right)\;|\;{\xi |}_{\Gamma }=0\right\}, \end{array}$$

where *ρ* ^*∞*^(*Ω*) is the set of functions infinitely many times differentiable on $\bar{\Omega }\in R^{2}$. Closure of set (43) in norm *H* ^2^(*Ω*) is a subspace in *H* ^2^(*Ω*) which is denoted by *V*(*Ω*). It is clear that *V*(*Ω*) = *H* ^2^(*Ω*)∩*H*_0_^1^(*Ω*).

It is known (see [13]) that a solution to problems (39) and (40) is equivalent to minimization on *V*(*Ω*) of the following functional:

(44) $$\begin{array}{c} J\left(v\right)=\frac{1}{2}\int_{\Omega }{\left|\Delta \xi \right|}^{2}d\Omega -\int_{\Omega }g\xi \;d\Omega -\frac{1}{2}\int_{\Gamma }\chi {\left|\frac{\partial \xi }{\partial n}\right|}^{2}ds. \end{array}$$

*Hybrid Variation Problem Formulation*. We assume that instead of functional (44) the following one is minimized:

(45) $$\begin{array}{c} \Phi \left(\xi ,\psi ,\alpha \right)=\frac{1}{2}\int_{\Omega }{\left|\psi \right|}^{2}d\Omega -\int_{\Omega }g\xi \;d\Omega -\frac{1}{2}\int_{\Gamma }\chi {\left|\alpha \right|}^{2}ds, \end{array}$$

on such triads (*ξ*, *ψ*, *α*) ∈ *V*(*Ω*) × *L* ^2^(*Ω*) × *L* ^2^(Γ) that their elements are coupled through equalities −Δ*ξ* = *ψ*, (∂*w*/∂*n*)|_Γ_ = *α*.

Let us define the space of the following functions:

(46) P(Ω)={(ξ,ψ,α)∈H01(Ω)×L2(Ω)×L2(Γ) ∣ ∀μ∈H1(Ω),β[(ξ,ψ,α),μ]=0},

where the bilinear form *β*[·, ·] is defined as

(47) $$\begin{array}{c} \beta \left[\left(\xi ,\psi ,\alpha \right),\mu \right]=\frac{1}{2}\int_{\Omega }\nabla \xi \nabla \mu \;d\Omega -\int_{\Omega }\psi \mu \;d\Omega -\int_{\Gamma }\alpha \mu \;ds. \end{array}$$

**Theorem 4**

If the space *Ω* is convex and has a Lipschitz continuous boundary Γ, then, first, the map (*ξ*, *ψ*, *α*) ∈ *P*(*Ω*) → |*ψ*|_0,*Ω*_^2^, now and later |*ξ*|_*m*,*Ω*_ = (∑_|*k*|=*m*_  ∫_*Ω*_|∂^*k*^ *ξ*|^2^ *dΩ*)^1/2^, ||*ξ*||_*m*,*Ω*_ = (∑_|*k*|≤*m*_  ∫_*Ω*_|∂^*k*^ *ξ*|^2^ *dΩ*)^1/2^) is the norm on space *P*(*Ω*) equivalent to the real dot product form (*ξ*, *ψ*, *α*) ∈ *P*(*Ω*) → (|*ξ*|_1,*Ω*_^2^+|*ψ*|_0,*Ω*_^2^+|*α*|_0,Γ_^2^)^1/2^ transforming *P*(*Ω*) into a Hilbert space; second, if (*ξ*, *ψ*, *α*) ∈ *P*(*Ω*), then

(48) $$\begin{array}{c} \left(\xi ,\psi ,\alpha \right)\in V\times L^{2}\left(\Omega \right)\times L^{2}\left(\Gamma \right),\;-\Delta \xi =\psi ,\;{\frac{\partial w}{\partial n}|}_{\Gamma }=\alpha , \end{array}$$

and if (48) is satisfied, then (*ξ*, *ψ*, *α*) ∈ *P*(*Ω*).

**Proof**

In the beginning we show the second statement. Since *Ω* has a continuous boundary, then the following Green formula holds:

(49) ∫Ω∇ξ∇μdΩ=−∫ΩΔξμdΩ+∫Γ∂ξ∂nμds,∀ξ∈H2(Ω),  ∀μ∈H1(Ω).

Let (*ξ*, *ψ*, *α*) ∈ *P*(*Ω*). Then *ξ* ∈ *H*_0_^1^(*Ω*), *ψ* ∈ *L* ^2^(*Ω*), *α* ∈ *L* ^2^(Γ), and *β*[(*ξ*, *ψ*, *α*), *μ*] = 0 for all *μ* ∈ *H* ^1^(*Ω*).

The last condition, in particular for ∀*μ* ∈ *H*_0_^1^(*Ω*) yields

(50) $$\begin{array}{c} \int_{\Omega }\nabla \xi \nabla \mu \;d\Omega =\int_{\Omega }\psi \mu \;d\Omega . \end{array}$$

It follows from (50) that *v* appears as a solution to the Dirichlet problem for the operator −Δ for *ξ*|_Γ_ = 0. Since space *Ω* is convex, therefore *ξ* ∈ *H* ^2^(*Ω*) ([13], Section 7.1, page 373), and consequently *ξ* ∈ *H* ^2^(*Ω*)∩*H*_0_^1^(*Ω*). Using now (50) for *μ* ∈ *H*_0_^1^(*Ω*), we get −Δ*ξ* = *ψ*. Using the Green formula for *μ* ∈ *H* ^1^(*Ω*), we find that (∂*ξ*/∂*n*)|_Γ_ = *α*. Assume that (48) holds. We show that (*ξ*, *ψ*, *α*) ∈ *P*(*Ω*). Since *ξ* ∈ *V*(*Ω*) ⊂ *H* ^2^(*Ω*) and −Δ*ξ* = *ψ*, (∂*ξ*/∂*n*)|_Γ_ = *α*, then (49) yields ∫_*Ω*_∇*ξ*∇*μ* *dΩ* = ∫_*Ω*_ *ψμ* *dΩ* + ∫_Γ_ *αμ* *ds* for all *μ* ∈ *H* ^1^(*Ω*); that is, *β*[(*ξ*, *ψ*, *α*), *μ*] = 0 for all *μ* ∈ *H* ^1^(*Ω*). Besides *ξ* ∈ *V*(*Ω*) ⊂ *H*_0_^1^(*Ω*) and the second statement is proved.

Let us prove the first statement. Endowed with the multiplication norm *P*(*Ω*) is a Hilbert space. Let (*ξ*, *ψ*, *α*) ∈ *P*(*Ω*). Then, as it has been shown, *ξ* ∈ *H* ^2^(*Ω*)∩*H*_0_^1^(*Ω*). For *μ* = *ξ* condition *β*[(*ξ*, *ψ*, *α*), *μ*] = 0 yields

(51) $$\begin{array}{c} {\left|\xi \right|}_{1,\Omega }^{2}\leq C_{1}{\left|\psi \right|}_{0,\Omega }{\left|\xi \right|}_{0,\Omega }. \end{array}$$

Let us introduce space *M* ⊂ *H* ^1^(*Ω*) such that *H* ^1^(*Ω*) = *H*_0_^1^(*Ω*) ⊕ *M*. Besides, let us introduce the following operator *B* : *H* ^1^(*Ω*) → *L* ^2^(*Ω*) defined in the following way: for all *ψ* ∈ *L* ^2^(*Ω*)  *α* = *Bψ* ∈ *L* ^2^(Γ) we have a unique solution to the following equation:

(52) $$\begin{array}{c} \int_{\Gamma }\alpha \mu \;ds=\int_{\Omega }\nabla \xi \nabla \mu \;d\Omega -\int_{\Omega }\psi \mu \;d\Omega \;\forall \mu \in M. \end{array}$$

Under the condition that *ξ* ∈ *H*_0_^1^(*Ω*) satisfies the following:

(53) $$\begin{array}{c} \int_{\Omega }\nabla \xi \nabla \mu \;d\Omega =\int_{\Omega }\psi \mu \;d\Omega \;\mu \in H_{0}^{1}\left(\Omega \right). \end{array}$$

It is not difficult to verify that under the theorem conditions, *Bψ* = −*B*Δ*ξ* = (∂*ξ*/∂*n*)|_Γ_; that is, *B* stands for the operator of external normal derivative for *ξ* ∈ *H* ^2^(*Ω*)∩*H*_0_^1^(*Ω*). This operator is bounded; that is, *ξ* ∈ *H* ^2^(*Ω*)∩*H*_0_^1^(*Ω*). We denote its norm by ||*B*||. Then ||*B*|| = sup⁡_*v*∈*H*^2^(*Ω*)∩*H*_0_^1^(*Ω*)_(||∂*ξ*/∂*n*||_0,Γ_/|Δ*ξ*|_0,*Ω*_), where ||•||_0, Γ_^  ^ denotes the norm associated with the scalar product (*α*,*β*)_*L*^2^(Γ)_ = ∫_Γ_ *αβ* *ds*. Therefore, for all *ψ* ∈ *L* ^2^(*Ω*)  ||*α*||_0,Γ_ ≤ ||*B*|||*ψ*|_0,*Ω*_. Hence, taking (52) into account we get (||*ξ*||_1,*Ω*_ + ||*ψ*||_0,*Ω*_ + ||*α*||_0,Γ_) ≤ C_2_||*ψ*||_0,*Ω*_ and the theorem is proved.

Results of this theorem allow us to transit from minimization of functional (45) on space *V*(*Ω*) to minimization of functional (45) on space *P*(*Ω*).

**Theorem 5**

Let *w* ∈ *V*(*Ω*) be a solution to problem (44), then

(54) $$\begin{array}{c} \Phi \left(w,-\Delta w,\frac{\partial w}{\partial n}\right)⟶\underset{\left(v,\psi ,\alpha \right)\in \underline{P}\left(\Omega \right)}{\inf}\Phi \left(\xi ,\psi ,\alpha \right). \end{array}$$

In this case the triad (*w*, −Δ*w*, ∂*w*/∂*n*) ∈ *P*(*Ω*) is the unique solution to the problem of minimization of (54).

**Proof**

We prove that a symmetric bilinear form *a*([(*ξ*, *ψ*, *α*), (*η*, *φ*, *β*)]) = ∫_*Ω*_ *ψφ* *dΩ* − ∫_Γ_ *χ* *αβ* *ds*,   (*ξ*, *ψ*, *α*), (*η*, *φ*, *β*) ∈ *P*(*Ω*) is continuous and elliptic on *P*(*Ω*).

Owing to Theorem 4, if (*ξ*, *ψ*, *α*), (*η*, *φ*, *β*) ∈ *P*(*Ω*), then −Δ*ξ* = *ψ*; (∂*ξ*/∂*n*)|_Γ_ = *α*; −Δ*η* = *φ*; (∂*η*/∂*n*)|_Γ_ = *β*. Then we have

(55) $$\begin{array}{c} a\left(\left[\left(\xi ,\psi ,\alpha \right),\left(\eta ,\phi ,\beta \right)\right]\right)=\int_{\Omega }\Delta \xi \Delta \eta \;d\Omega -\int_{\Gamma }\frac{\partial \xi }{\partial n}\frac{\partial \eta }{\partial n}ds. \end{array}$$

For *η* = *ξ*, *φ* = *ψ*, *β* = *α* from (55) we obtain ([13], Section 1.2, page 38)

(56) a([(ξ,ψ,α),(η,ψ,α)])= ∫Ω[(∂2ξ∂x2)2+2∂2ξ∂x2∂2ξ∂y2+(∂2ξ∂x2)2+2(∂2ξ∂x ∂y)2]dΩ≥|ψ|0,Ω2.

Then, elliptic property has been proved by *P*. Continuity of the bilinear form is evident. It means that the problem of minimization

(57) $$\begin{array}{c} \Phi \left(\xi^{*},\psi^{*},\alpha^{*}\right)⟶\underset{\left(u,\varphi ,\beta \right)\in P\left(\Omega \right)}{\inf}\Phi \left(\eta ,\varphi ,\beta \right) \end{array}$$

has a solution which is unique. Let us find a link between a solution to problem (37) as well as problems (41) and (42). If (ξ*, ψ*, α*) ∈ *P*(*Ω*) is a solution to problem (57), then the following relations should hold:

(58) ∫Ωψ∗φ dΩ−∫Ωηg dΩ−∫Γχα∗β ds=0∀(η,φ,β)∈P(Ω).

Since (ξ*, ψ*, α*) ∈ *P*(*Ω*), then −Δξ* = ψ*, (∂ξ*/∂*n*)|_Γ_ = α*, and ξ* ∈ *P*(*Ω*). Therefore, taking (58) into account we get ∫_*Ω*_Δξ*Δ*η* *dΩ* − ∫_Γ_ *χ*(∂ξ*/∂*n*)(∂*η*/∂*n*) *ds* = ∫_Γ_ *η* *g* *ds*. Therefore, ξ* coincides with the solution *w* to problems (39) and (40), and ψ* = −Δ*w*, α* = (∂*w*/∂*n*)|_Γ_.

**Remark 6**

Since the space is convex and its boundary is regular, then for *g* ∈ *H* ^−1^(*Ω*) a solution to problems (39) and (40),

(59) $$\begin{array}{c} w\in H^{3}\left(\Omega \right)\cap H_{0}^{1}\left(\Omega \right),\;\;\Delta w\in H^{1}\left(\Omega \right). \end{array}$$

*Solution to the Minimization Problem (44).* We show that a solution to problem (44) can be reduced to a solution of successive Dirichlet problems for the operator −Δ.

For further analysis it is suitable to introduce a linear transformation *A* : *L* ^2^(*Ω*) → *H*_0_^1^(*Ω*) in the following way: if *ψ* ∈ *L* ^2^(*Ω*) is a given function, then the function *ξ* = *Aψ* ∈ *H*_0_^1^(*Ω*) is a unique solution to the equation ∫_*Ω*_∇*ξ*∇*μ* *dΩ* = ∫_*Ω*_ *ψμ* *dΩ*  ∀ *μ* ∈ *H*_0_^1^(*Ω*), for *ξ* ∈ *H*_0_^1^(*Ω*). This means that space *P*(*Ω*), defined by (43), can be presented in the following form:

(60) P(Ω)={(ξ,ψ,α)∈H01(Ω)×L2(Ω)×L2(Γ) ∣ ξ=Aψ,  α=Bψ}.

Problem (44) is equivalent to the following problem of optimal control:

(61) $$\begin{array}{c} \underset{\psi \in L^{2}\left(\Omega \right)}{\min}\left[\frac{1}{2}\int_{\Omega }{\left|\psi \right|}^{2}d\Omega -\int_{\Omega }g\xi \;d\Omega -\frac{1}{2}\int_{\Gamma }\chi {\left|\alpha \right|}^{2}ds\right], \end{array}$$

where the state function *ξ* and *α* are coupled via control *ψ* ∈ *L* ^2^(*Ω*) through the following state equations:

(62) $$\begin{array}{c} \xi \in H_{0}^{1}\left(\Omega \right),\;\xi =A\psi ,\\ \alpha \in L^{2}\left(\Gamma \right),\;\alpha =B\psi . \end{array}$$

As it follows from Remark 6, although the optimal control *ψ* is sought on *L* ^2^(*Ω*), its regularity is higher for *g* ∈ *H* ^−1^(*Ω*) *ψ* ∈ *H* ^1^(*Ω*). In this case the following trace is defined: *ψ*|_Γ_ = *λ*, *λ* ∈ *M*. Furthermore, besides (62), we require that ∫_*Ω*_∇*ψ*_*λ*_∇*μ* *dΩ* = ∫_*Ω*_ *gμ* *dΩ* for all *μ* ∈ *H*_0_^1^(*Ω*) and *ψ*_*λ*_ − *λ* ∈ *H*_0_^1^(*Ω*). Then, if *ξ*_*λ*_ = *Aψ*_*λ*_ and *α*_*λ*_ = *Bψ*_*λ*_, (61) implies that min⁡_*ψ*∈*L*^2^(*Ω*)_Φ(*ξ*, *ψ*, *α*) = min⁡_*λ*∈*M*_ *D*(*λ*), where

(63) D(λ)=−12∫Ω|ψλ|2dΩ−∫Γλαλ ds−12∫Γχ|αλ|2ds ∀λ∈M.

The fundamental idea consists now in the application of a gradient method to the problem of minimization (63).

Let us take *M*′ as a dual space for space *M*, and let 〈·, ·〉 denote the relation of duality between spaces *M* and *M*′. We denote by *D*′ ∈ *M*′ a derivative of the functional *D*(*λ*). Let us introduce a map *S* : *M* → *H* ^1^(*Ω*) in the following way: for $\lambda \in M\;\;\overset{\circ }{\varphi_{\lambda }}=S\left(\lambda \right)$ is a unique function from *H* ^1^(*Ω*) satisfying the condition

(64) $$\begin{array}{c} \int_{\Omega }\nabla {\overset{\circ }{\phi }}_{\lambda }\nabla \mu \;d\Omega =0,\;\forall \mu \in H_{0}^{1}\left(\Omega \right),\;\;\\ {\overset{\circ }{\phi }}_{\lambda }-\lambda \in H_{0}^{1}\left(\Omega \right). \end{array}$$

**Theorem 7**

For an arbitrary *λ* ∈ *M* defined in (63), the functional is differentiable and its derivative is defined by the relation

(65) ∀μ∈M 〈D′(λ),μ〉=∫Ωφ∘θφ∘μ dΩ,  where  φ∘μ=S(μ),  φ∘θ=S(θ  λ  ),θλ=λ+χαλ, θλ∈M.

**Proof**

Differentiating (63) yields

(66) 〈D′(λ),μ〉=−∫Ωψλϕ∘μdΩ−∫Γαλμ dΩ−∫Γ(λ+χ  αλ)β∘μds,

where ${\overset{\circ }{\varphi }}_{\mu }=S\left(\mu \right),\;{\overset{\circ }{\beta }}_{\mu }=B{\overset{\circ }{\varphi }}_{\mu }$.

From (66) and taking (52) into account we get

(67) $$\begin{array}{c} \langle D^{\prime }\left(\lambda \right),\mu \rangle =-\int_{\Omega }\nabla v_{\lambda }\nabla {\overset{\circ }{\phi }}_{\mu }d\Omega -\int_{\Gamma }\left(\lambda +\chi \alpha_{\lambda }\right){\overset{\circ }{\beta }}_{\mu }ds. \end{array}$$

First term in (67) is equal to zero due to (64). Let us introduce the function ${\overset{\circ }{\varphi }}_{\theta }=S\left(\theta_{\lambda }\right)$, where *θ*_*λ*_ is defined by (66) and let ${\overset{\circ }{u}}_{\mu }=A{\overset{\circ }{\varphi }}_{\mu }$. Then, (66) implies $\langle D^{\prime }(\lambda ),\mu \rangle =\int_{\Omega }{\overset{\circ }{\phi }}_{\theta }{\overset{\circ }{\phi }}_{\mu }d\Omega -\int_{\Gamma }\nabla {\overset{\circ }{\phi }}_{\theta }\nabla {\overset{\circ }{u}}_{\mu }d\Omega$.

However, the last term in this equality is equal to zero due to (64), which ends the proof. The gradient method applied to minimization (63) consists now in the determination of a series {*λ*_*n*_}_*n*=0_^*∞*^ of functions *λ* ^*n*^ ∈ *M* via the following iteration scheme:

(68) $$\begin{array}{c} \mu \in M\;{\left(\lambda^{n+1}-\lambda^{n},\mu \right)}_{M}=-\rho_{n}\langle D^{\prime }\left(\lambda^{n}\right),\mu \rangle , \end{array}$$

where (·,·)_*M*_ is the arbitrary scalar product in space *M*, *ρ* is the positive parameter, and *λ* ^0^ is the arbitrary function of *M*.

Therefore, one iteration (68) corresponds to successive solutions of the following problems:

(a) find for a given function *λ* ^*n*^ ∈ *M* a unique function *ψ* ^*n*^ ∈ *H* ^1^(*Ω*), satisfying the following relations:
  (69) $$\begin{array}{c} \psi^{n}-\lambda^{n}\in H_{0}^{1}\left(\Omega \right), \end{array}$$
  (70) $$\begin{array}{c} \forall \mu \in H_{0}^{1}\left(\Omega \right)\;\int_{\Omega }\nabla \psi^{n}\nabla \mu \;d\Omega =\int_{\Omega }g\mu \;d\Omega ; \end{array}$$
(b) find a function *ξ* ^*n*^ ∈ *H*_0_^1^(*Ω*), satisfying the following relation:
  (71) $$\begin{array}{c} \forall \mu \in H_{0}^{1}\left(\Omega \right)\;\int_{\Omega }\nabla \xi^{n}\nabla \mu \;d\Omega =\int_{\Omega }\psi^{n}\mu \;d\Omega ; \end{array}$$
(c) find a function *α* ^*n*^ ∈ *L* ^2^(Γ), satisfying the following relation:
  (72) $$\begin{array}{c} \forall \mu \in M\;\int_{\Gamma }\alpha^{n}\mu \;d\Omega =\int_{\Omega }\nabla \xi^{n}\nabla \mu \;d\Omega \;-\int_{\Omega }\psi^{n}\mu \;d\Omega ; \end{array}$$
(d) find a function *λ* ^*n*+1^ ∈ *M*, satisfying the following relation:
  (73) $$\begin{array}{c} \forall \mu \in M\;{\left(\lambda^{n+1}-\lambda^{n},\mu \right)}_{M}=-\rho \int_{\Omega }\overset{\circ }{\phi_{\theta }^{n}}\overset{\circ }{\phi_{\mu }}d\Omega ; \end{array}$$

where $\overset{\circ }{\varphi_{\theta }^{n}}=S\theta_{\lambda }^{n}$, *θ*_*λ*_^*n*^ = *λ* ^*n*^ − *χ* *Bψ* ^*n*^, and $\overset{\circ }{\varphi_{\mu }}=S\mu$.

We show that by a proper choice of parameter *ρ* > 0 the iteration process ((69)–(73)) is convergent for arbitrarily taken initial approximation.

Let us first define the map *C* : *H* ^1^(*Ω*) → *M* in the following way: for any function *ψ* ∈ *H* ^1^(*Ω*) function *Cψ* ∈ *M* is unique satisfying the following condition:

(74) $$\begin{array}{c} \forall \mu \in M\;{\left(C\psi ,\mu \right)}_{M}=\int_{\Omega }\psi \overset{\circ }{\phi_{\mu }^{}}d\Omega . \end{array}$$

Let us take ||*C*|| = sup⁡_*ψ*∈*H*^1^(*Ω*)_(|*Cψ*|_*μ*_/|*ψ*|_0,*Ω*_), where |·|_*M*_ denotes the norm associated with the scalar product (·,·)_*M*_. It is clear that this norm exists, since the map $\mu \in M\to {\overset{\circ }{\varphi }}_{\mu }\in H^{1}\left(\Omega \right)$ is bounded.

**Theorem 8**

If parameter *ρ* satisfies the following condition:

(75) $$\begin{array}{c} 0<\rho <\frac{2}{{||C||}^{2}{\left(1-{\left|\chi \right|}_{L^{\infty }(\Omega )}||S||||B||\right)}^{2}}, \end{array}$$

then the iteration process (69)–(73) is convergent in the sense that

(76) $$\begin{array}{c} \underset{n\to \infty }{\lim}\psi^{n}=\psi \;\;в\;\;L^{2}\left(\Omega \right),\;\;\underset{n\to \infty }{\lim}\xi^{n}=\xi \;\;в\;\;H_{0}^{1}\left(\Omega \right),\\ \underset{n\to \infty }{\lim}\alpha^{n}=\alpha \;\;в\;\;L^{2}\left(\Gamma \right). \end{array}$$

**Proof**

It is sufficient to show that lim⁡_*n*→*∞*_ *ψ* ^*n*^ = 0 in *L* ^2^(*Ω*) in the particular case when *g* = 0. If we use the definition (74) of map *C*, then the recurrent formula (73) gives

(77) $$\begin{array}{c} \lambda^{n+1}=\lambda^{n}-\rho C\overset{\circ }{\varphi_{\theta }^{n}},\;\text{where}\;\;\overset{\circ }{\varphi_{\theta }^{n}}=S\theta_{\lambda }^{n},\;\;\theta_{\lambda }^{n}=\lambda^{n}+\chi B\psi^{n}, \end{array}$$

and therefore

(78) $$\begin{array}{c} {\left|\lambda^{n+1}\right|}_{M}^{2}={\left|\lambda^{n}\right|}_{M}^{2}-2\rho {\left(C\overset{\circ }{\phi_{\theta }^{n}},\lambda^{n}\right)}_{M}+\rho^{2}{\left|C\overset{\circ }{\phi_{\theta }^{n}}\right|}_{M}^{2}. \end{array}$$

Consider the term ${\left(C\overset{\circ }{\varphi_{\theta }^{n}},\lambda^{n}\right)}_{M}$:

(79) (Cφθn∘,λn)M=∫Ωφθn∘ψndΩ=−∫Γ(λn+χBψn)Bψnds=∫Ω|ψn|2dΩ−∫Γχ|Bψn|2ds≥|ψn|0,Ω2.

Let us estimate the norm $\left|\overset{\circ }{\varphi_{\theta }^{n}}\right|$:

(80) |φθn∘|0,Ω=|S(λn+χBψn)|0,Ω≤|ψn|0,Ω +||S|||χ|L∞(Ω)||B|||ψn|0,Ω≤(1+|χ|L∞(Ω)||S||||B||)  |ψn|0,Ω.

The latter inequalities and (78) imply the following estimation:

(81) |λn+1|M2−|λn|M2  ≤−ρ[2η−||C||2(1+|χ|L∞(Ω)||S||||B||  )2ρ]|ψn|0,Ω2.

Hence, in particular, we get

(82) $$\begin{array}{c} \underset{n\to \infty }{\lim}{\left|\psi \right|}_{0,\Omega }^{n}=0, \end{array}$$

if *ρ* satisfies inequalities (75). Besides, we have

(83) $$\begin{array}{c} \underset{n\to \infty }{\lim}\xi^{n}=\underset{n\to \infty }{\lim}A\psi^{n}=0\;\;в\;\;H_{0}^{1}\left(\Omega \right),\\ \underset{n\to \infty }{\lim}\alpha^{n}=\underset{n\to \infty }{\lim}B\psi^{n}=0\;\;в\;\;L^{2}\left(\Gamma \right), \end{array}$$

which finishes the proof.

Since convergence of the considered method is guaranteed, any choice of subspace *M*, satisfying the condition *H* ^1^(*Ω*) = *H*_0_^1^(*Ω*) ⊕ *M* and any choice of scalar product (·,·)_*M*_ on space is allowed. However, the choice influences parameter *ρ*, as well as the computation time on each iterations. Finally, we point out a few remarks regarding practical computations of *λ* ^*n*+1^ ∈ *M*.

If the scalar product in *M* is defined via the following formula:

(84) $$\begin{array}{c} {\left(\lambda ,\mu \right)}_{M}=\int_{\Omega }S\lambda \cdot S\mu \;d\Omega , \end{array}$$

then as *λ* ^*n*+1^ any function from *M* can be taken, assuming that the following condition is satisfied:

(85) $$\begin{array}{c} {\lambda^{n+1}|}_{\Gamma }={\left(\lambda^{n}-\rho \theta_{\lambda }^{n}\right)|}_{\Gamma }. \end{array}$$

Equality (85) can be understood in the sense of trace equality on a boundary. In fact, if (85) is satisfied, then

(86) $$\begin{array}{c} {\left(\lambda^{n+1}-\lambda^{n},\mu \right)}_{M}=-\rho \int_{\Omega }S\theta^{n}\cdot S\mu \;d\Omega =-\rho \int_{\Omega }\overset{{}^{\circ}}{\varphi_{\theta }^{n}}\overset{\circ }{\varphi_{\mu }}d\Omega , \end{array}$$

which means that conditions of Theorem 7 are satisfied.

We may choose also the following scalar product:

(87) $$\begin{array}{c} \text{either}\;\;{\left(\lambda ,\mu \right)}_{M}=\int_{\Omega }\nabla \lambda \nabla \mu \;d\Omega .\\ \text{or}\;\;{\left(\lambda ,\mu \right)}_{M}=\int_{\Omega }\lambda \mu \;d\Omega . \end{array}$$

However, in the latter case one needs to compute gradient 〈*D*′(*λ*), *μ*〉 on each step, which extends the computational time.

*Final Remarks*. (1) The proof has been carried out for equations in the hybrid form (2). It can be relatively easily extended into equations regarding displacements. (2) Results can be extended on other types of the differential equations, including nonlinear ones, consisting of a biharmonic operator.

### 3.3. Iterative Procedure for the Reduction of the Karman Equation into the Poisson Equation

In the preceding sections we have proved convergence of the iterative procedures for linearization of (2) by reducing the solution of the eighth order system of nonlinear differential equations into that of the solution to a biharmonic equation, as well as the reduction of the biharmonic equation to the Poisson-type equation in the case of a curvilinear boundary using the finite element method (FEM).

While considering a space with the rectangular boundary, we may extend the procedure reported in Section 3.1 by introduction of new variables into the iterative procedure of solution to the Poisson-type equations without difficulties.

In the case of spaces with the curvilinear boundary, the procedure described in Section 3.2 can be applied to solve (2) using the iterative procedure, whose convergence has been proved in Section 3.1.

For this purpose new variables *M*_*w*_(*x*, *y*) and *M*_*F*_(*x*, *y*) are introduced

(88) $$\begin{array}{c} M_{w}\left(x,y\right)=\Delta w\left(x,y\right),\;\;M_{F}\left(x,y\right)=\Delta F\left(x,y\right). \end{array}$$

Then each of differential equations (2) is divided into two Poisson-type equations. The iterative procedure of solution of the obtained system of four Poisson-type equations has the following form:

(89) $$\begin{array}{c} \Delta M_{w}^{\left(k\right)}=q+L\left(w^{\left(k-1\right)},F^{\left(k-1\right)}\right),\\ \Delta w^{\left(k\right)}=M_{w}^{\left(k\right)},\\ \Delta M_{F}^{\left(k\right)}=L\left(w^{\left(k\right)},w^{\left(k\right)}\right),\\ \Delta F^{\left(k\right)}=M_{F}^{\left(k\right)},\;\left\{x,y\right\}\in \Omega . \end{array}$$

Boundary conditions (4) are transformed to the following form:

(90) $$\begin{array}{c} {w|}_{\Gamma }={M_{w}|}_{\Gamma }={F|}_{\Gamma }={M_{F}|}_{\Gamma }=0. \end{array}$$

The given procedure (89) has advantages over procedure (6), while solving each equation since instead of the fourth order equation that of the second order is solved. Because equations are solved by numerical methods (FDM, FEM) and the approximation of the biharmonic operator has high requirements on the approximating functions, then for the Poisson-type equation one may simplify the procedure (89) of finding a solution by choosing simple approximating functions.

In the FDM case, an order of algebraic equations system, after a discretization of the biharmonic equation, is higher for the second order equation, and hence higher expectations are required from computer abilities while solving the problem numerically.

## 4. The Method of Variational Iterations (MVI) of PDEs Solutions

### 4.1. Validation of Convergence

The method of variational iterations (MVI) was applied first in 1933 by Shunok who considered a deflection of cylindrical panels. However, this work did not meet with the response of others, and then it was rediscovered in the sixties of the previous century by Kantorovich and Krylov [20], who applied it in his investigation of rectangular plates. Then the MVI found wide application in solving various problems of plates and shells (see the list of references reported in [21]).

Here we prove validity and reliability of the mentioned method for a class of equations with positively defined operators, that is, biharmonic and harmonic ones. In other words, we prove a theorem on convergence of the MVI for iterative procedures (6) and (89).

Formally, the MVI scheme is as follows. Assume that our aim is to find a solution to the following:

(91) $$\begin{array}{c} T\omega \left(x,y\right)=g\left(x,y\right),\;\;x,y\in \Omega \left(x,y\right), \end{array}$$

where *T* stands for a certain operator defined on set *D*(*T*) of the Hilbert space *L*_2_(*Ω*); *g*(*x*, *y*) is the function given for two variables *x* and *y*; *ω*(*x*, *y*) is the function of these two variables being sought; *Ω*(*x*, *y*) is the space of changes of variables *x* and *y*.

If *Ω*(*x*, *y*) = *X* × *Y* (*X* is the certain bounded set of variables *x*, *Y* is the bounded set of *y*), then a solution to (91) can be given in the following form:

(92) $$\begin{array}{c} \omega_{N}\left(x,y\right)=\overset{N}{\underset{i=1}{\sum }}u_{i}\left(x\right)v_{i}\left(y\right), \end{array}$$

where functions *u*_*i*_(*x*) and *v*_*i*_(*y*) are defined by the following system of equations:

(93) $$\begin{array}{c} \overset{}{\underset{X}{\int }}\left(T\omega_{N}-g\right)u_{1}\left(x\right)dx=0,\\ \vdots \\ \overset{}{\underset{X}{\int }}\left(T\omega_{N}-g\right)u_{N}\left(x\right)dx=0,\\ \overset{}{\underset{Y}{\int }}\left(T\omega_{N}-g\right)v_{1}\left(y\right)dy=0,\\ \vdots \\ \overset{}{\underset{Y}{\int }}\left(T\omega_{N}-g\right)v_{N}\left(y\right)dy=0. \end{array}$$

It is found in the following way: we have a certain system composed of *N* functions regarding one variable, for instance, *u*_1_^0^(*x*), *u*_2_^0^(*x*),…, *u*_*N*_^0^(*x*), and then from the first *N* equations of system (93) the system of *N* functions *v*_1_^1^(*x*), *v*_2_^1^(*x*),…, *v*_*N*_^1^(*x*) is defined. Then, the so far obtained functions represent a new choice of the functions regarding the variable *x* − *u*_1_^2^(*x*), *u*_2_^2^(*x*),…, *u*_*N*_^2^(*x*), and the latter serves to get a new set of functions regarding variable *y* − *v*_1_^3^(*x*), *v*_2_^3^(*x*),…, *v*_*N*_^3^(*x*), and so forth.

**Definition 9**

We say that a process of computation, when one given system of functions is replaced by the second system, is the MVI step. The number of steps needed to define a certain choice of functions corresponds to the superscript (number) of functions being considered. Truncating the process of finding functions *u*_*i*_(*x*) and *v*_*i*_(*y*) on the *k*th step, which, for example, corresponds to the choice of functions *v*_1_^*k*^(*y*), *v*_2_^*k*^(*y*),…, *v*_*N*_^*k*^(*y*), we define the function

(94) $$\begin{array}{c} \omega_{N}^{k}=\overset{N}{\underset{i=1}{\sum }}u_{i}^{k-1}\left(x\right)v_{i}^{k}\left(y\right), \end{array}$$

taken as the approximating solution of (91) obtained by MVI.

**Remark 10**

Here and further on, we shall take as operator *T* a certain differential operator defined on set *D*(*T*) of the Hilbert space *L*_2_(*Ω*). Then, on each step system (93) shall be transformed to a system of ODEs which can be solved further.

**Remark 11**

We call function *ω*_*N*_(*x*, *y*) the *N*th approximation to (91) if the number of series terms in (92) is equal to *N*.

Let us study the case of first approximation; that is, the following solution of (91) is sought:

(95) $$\begin{array}{c} \omega_{1}\left(x,y\right)=u\left(x\right)v\left(y\right), \end{array}$$

where functions *u*(*x*) and *v*(*y*) are defined through the illustrated way from the following system of equations:

(96) $$\begin{array}{c} \int_{X}\left(Tu\left(x\right)\cdot v\left(y\right)-g\right)u\left(x\right)dx=0,\\ \int_{Y}\left(Tu\left(x\right)\cdot v\left(y\right)-g\right)u\left(y\right)dy=0. \end{array}$$

Let the operator *T* in (91) be positive definite. Let us introduce the following notation: *H*_*T*_(*X* × *Y*) is the energy space of the operator *T*; [·, ·] is the scalar product of elements in *H*_*T*_; *ω*_0_ is the exact solution to (91).

**Theorem 12**

If *T* is a positive definite operator with the space of action *D*(*T*) ⊂ *H*_*T*_, then the sequence of elements *α*_*k*_ = ||*ω*_1_^*k*^(*x*,*y*)−*ω*_0_||_*H*_*T*__ is monotonously decreasing; that is, for arbitrary *i* and *j* if *i* ≥ *j*, then

(97) $$\begin{array}{c} {||\omega_{1}^{i}-\omega_{0}||}_{H_{T}}\leq {||\omega_{1}^{j}-\omega_{0}||}_{H_{T}}. \end{array}$$

**Proof**

We consider a subset *M*_1_^1^ of the space *H*_*T*_ which has the following form:

(98) M11={ω(x,y) ∣ ω(x,y)=u0(x)v(y),u0(x)∈HT(x),v(y)∈HT(Y)}.

It is clear, that set *M*_1_^1^ represents a subspace of space *H*_*T*_(*X* × *Y*) (generally, of infinite dimension). Therefore, one may define *ω*_0_ projection onto space *M*_1_^1^. As it is known that element *u* ^0^(*x*)v*(*y*) ∈ *M*_1_^1^ stands for the projection of *ω*_0_ onto *M*_1_^1^ if the following condition is satisfied:

(99) $$\begin{array}{c} {\left[u^{0}\left(x\right)v^{*}\left(y\right)-\omega_{0},u^{0}\left(x\right)v\left(y\right)\right]}_{H_{T}}=0 \end{array}$$

for arbitrary elements *u*_0_(*x*)*v*(*y*) ∈ *M*_1_^1^. It is clear that if *u* ^0^(*x*)v*(*y*) ∈ *M*_1_^1^, then (99) coincides with the first equation of system (97).

Since the element *u* ^0^(*x*)*v* ^1^(*y*) obtained through the first step of MVI is a projection of element *ω*_0_ onto the subspace *M*_1_^1^, hence the following inequality holds:

(100) $$\begin{array}{c} {||\omega_{1}^{1}\left(x,y\right)-\omega_{0}||}_{H_{T}}\leq {||u^{0}\left(x\right)v\left(y\right)-\omega_{0}||}_{H_{T}} \end{array}$$

for arbitrary elements *u* ^0^(*x*)*v*(*y*) ∈ *M*_1_^1^. An analogous construction allows us to get a similar inequality for the subspaces; that is, we have

(101) M21={ω(x,y) ∣ ω(x,y)=u0(x)v1(y),u(x)∈HT(x),v1(y)∈HT(Y)}.

In the case corresponding to the second MVI step,

(102) $$\begin{array}{c} {||u^{2}\left(x\right)v^{1}\left(y\right)-\omega_{0}||}_{H_{T}}\leq {||u\left(x\right)v^{1}\left(y\right)-\omega_{0}||}_{H_{T}} \end{array}$$

for arbitrary elements *u*(*x*)*v* ^1^(*y*) ∈ *M*_2_^1^. It follows from (100) and (102) that ||*u* ^2^(*x*)*v* ^1^(*y*)−*ω*_0_||_*H*_*T*__ ≤ ||*u* ^0^(*x*)*v* ^1^(*y*)−*ω*_0_||_*H*_*T*__. Considerations similar to those so far provided and obtained for the *k*th MVI step prove the theorem as well as inequality (97) with the help of induction.

**Remark 13**

Results of Theorem 12 are extended into the case of *N*th approximation, and therefore inequality (97) is given in the following form:

(103) $$\begin{array}{c} {||\omega_{N}^{n}\left(x,y\right)-\omega_{0}||}_{H_{T}}\leq {||\omega_{N}^{n}\left(x,y\right)-\omega_{0}||}_{H_{T}},\;m\geq n. \end{array}$$

In order to prove the theorem we introduce the following lemma.

**Lemma 14**

Let each of elements of the basis system of space *H*_*T*_ have the following form:

(104) $$\begin{array}{c} \theta_{i}\left(x,y\right)=\varphi_{i}\left(x\right)\psi_{i}\left(y\right),\;\forall^{i}\varphi_{i}\left(x\right)\in H_{T}\left(X\right),\;\;\varphi_{i}\left(y\right)\in H_{T}\left(Y\right). \end{array}$$

If for the initial MVI approximation one takes any component of a certain basis function *θ*_*i*_, that is, *u* ^0^(*x*) ≡ *φ*_*i*_(*x*), then for an arbitrary number *k* of the MVI steps the following inequality holds:

(105) $$\begin{array}{c} {||\omega_{1}^{k}\left(x,y\right)-\omega_{0}||}_{H_{T}}\leq {||c\varphi_{i}\left(x\right)\psi_{i}\left(y\right)-\omega_{0}||}_{H_{T}}, \end{array}$$

where *c* is the arbitrarily taken real number.

**Proof**

Since *u* ^0^(*x*)*v* ^1^(*y*) ≡ *φ*_*i*_(*x*)*v* ^1^(*y*), then Theorem 12 yields

(106) ||ω1k(x,y)−ω0||HT≤||φi(x)v1(y)−ω0||HT≤||cφi(x)ψi(y)−ω0||HT.

On the basis of the given lemma we formulate one of the MVI convergence criterions. Initially, we identify space *H*_*T*_ with space $\overset{0}{W_{2}^{m}}(\Omega )$ which is generated through a closure regarding the norm

(107) $$\begin{array}{c} {||\omega ||}_{W_{2}^{m}}={\left\{\overset{}{\underset{\Omega }{\int }}\overset{m}{\underset{k=0}{\sum }}\;\overset{}{\underset{\left(k\right)}{\sum }}\left|\frac{\partial^{k}\omega }{\partial^{k_{1}}x\partial^{k_{2}}y}\right|dx\;dy\right\}}^{2} \end{array}$$

of a set of infinitely differentiable functions $\overset{0}{C^{\infty }}(\Omega )$ with a compact carrier in  *Ω*.

**Theorem 15**

Let each of elements of the basis system of space $\overset{0}{W_{2}^{m}}(X\times Y)$ have the following form:

(108) $$\begin{array}{c} \theta_{i}\left(x,y\right)=\varphi_{i}\left(x\right)\psi_{i}\left(y\right), \end{array}$$

where {*φ*_*i*_(*x*)} stands for the basis system in space $\overset{0}{W_{2}^{m}}(X)$ and {*ψ*_*i*_(*y*)} in space $\overset{0}{W_{2}^{m}}(Y)$, and in order to get an arbitrary *N*th order approximation of the MVI we take components of the elements of the basis system {*θ*_*i*_(*x*, *y*)} as the initial functions. Then, for sufficiently large *N* the MVI gives a unique approximate solution *ω*_*N*_, and the sequence {*ω*_*N*_} is convergent with respect to the norm of space $\overset{0}{W_{2}^{m}}(X\times Y)$ to the exact solution *ω*_0_ irrespectively of the number of steps *k*. This construction can be carried out for each *N*th approximation; that is,

(109) $$\begin{array}{c} {||\omega_{N}^{k}-\omega_{0}||}_{\overset{0}{W_{2}^{m}}}⟶0,\;N⟶\infty . \end{array}$$

**Proof**

If we prove the theorem regarding approximations obtained via the first step of MVI, then owing to the Lemma the obtained results shall be valid for the arbitrary *k*th step. Therefore, let us consider the *N*th approximation of problem (91), obtained on the first step. In a way similar to considerations regarding Theorem 12, one may show that each *ω*_*N*_^1^ stands for a projection of element *ω*_0_ onto the following subspace:

(110) $$\begin{array}{c} M_{N}^{1}=\left\{\omega \left(x,y\right)\;|\;\omega \left(x,y\right)=\overset{N}{\underset{i=1}{\sum }}u^{0}\left(x\right)v^{1}\left(y\right)\right\}, \end{array}$$

where *u*_*i*_^0^(*x*) denotes *N* the fixed elements from system {*φ*_*i*_(*x*)}, and for arbitrary *i* and *v*_*i*_(*y*) they cover the whole space $\overset{0}{W_{2}^{m}}(Y)$. Therefore, *ω*_*N*_^1^ = *P*_*N*_ *ω*_0_, where *P*_*N*_ stands for the operator of an orthogonal projection onto subspace *M*_*N*_^1^ which is bounded. Since elements of the basis system *θ*_*i*_(*x*, *y*) have form (108), it is limiting dense ([20], page 191) in $\overset{0}{W_{2}^{m}}(X\times Y)$. Then the proof is carried out in a way similar to that of Theorem 16.2 (see [20], page 216), since all conditions of its application are satisfied.

**Remark 16**

Results of Theorem 12 and the Lemma indicate that using the MVI one may get an approximate solution to (91) in a way not worse than that obtained via the Ritz method in accordance with the corresponding subspace.

The MVI can be extended also on the case of a large number of variables. For instance, a sought solution to (91) is the function of three variables *x*, *y*, *z*, and hence the approximate solution of MVI can be sought in the following form:

(111) $$\begin{array}{c} \omega \left(x,y,z\right)=u\left(x\right)v\left(y,z\right). \end{array}$$

**Remark**

Note that during application of the MVI there is no need to construct the initial condition, satisfying, say, boundary conditions of the stated problem. Let us assume that operator *T* defines a certain boundary value problem. Let us introduce an arbitrary function from a space of the definition of the differential operator of the studied problem. Then on the first (second) step we get a system of functions satisfying boundary conditions regarding one (two) of the variables.

Observe that the MVI, on each step, defines only one of the functions appearing in the representation of solution (108). The following method develops MVI and allows us, using only a first step, to estimate at once two functions regarding two directions of the coordinates.

### 4.2. Numerical Results

Let us consider the following rectangular plate

(112) $$\begin{array}{c} \Delta^{2}\omega =\frac{g\left(x,y\right)}{D}, \end{array}$$

where *ω*(*x*, *y*) is the normal plate deflection in point *x*, *y*; *g*(*x*, *y*) is the intensity of the normal load; *D* = *Eh* ^3^[12(1 − *ν* ^2^)]^−1^; *E*, *ν* are the Young modulus and Poisson constant, respectively; 2*h* is the plate thickness, $\overset{-}{y}=0$; $1,\overset{-}{x}=0;\;1$, where $\overset{-}{y}=y/b$, $\overset{-}{x}=x/a$; *a* and *b* are the plate dimensions; and $\overset{-}{g}(x,y)=ga^{2}b^{2}{\left[Eh^{4}\right]}^{-1}$. Plate space in the plane *x*, *y* is denoted by *Ω*, and its contour is Γ. Below, we study two types of boundary conditions.

In the case of a simple support *w* = ∂^2^ *w*/∂*n* ^2^|_Γ_ = 0, a solution obtained via variational iterations is compared with that obtained through the first order approximation of the Bubnov method and with the solution represented by double trigonometric series. The following deflection function has been assumed: *W*(*x*, *y*) = *A*sin(*πx*)sin(*πy*), which satisfies the boundary conditions. Substituting this into (112) and applying the Bubnov procedure we obtain *A* = (4/*π* ^6^)*q*, and for *q* = 1 we have *A* = 0.00416. The deflection function *w*(*x*, *y*) = ∑_*m*,*n*_ *A*_*m*,*n*_sin⁡(*m* *πx*/*a*)sin⁡(*n* *πy*/*b*) is substituted into (20) then multiplied by sin⁡(*m* *πx*/*a*), sin⁡(*n* *πy*/*b*), and integrated regarding the plate surface. The following deflection value is obtained:

(113) w(x,y)=q24D(x4−2ax3+a3x) −4qa4π5Dsinmπxa ×∑m,n1m5[amth(αm)+22ch(αm)ch(2αmyb)−αm2ch(αm)2ybsh(2αmyb)].

It should be emphasized that the obtained series converges fast, and in practice it is sufficient to keep only its first term. For the square plate, the deflection measured in its center is 0.00406 (Table 1).

In the second case the plate contours are clamped; that is, *w* = (∂*w*/∂*n*)|_Γ_ = 0. Here computations are carried out in the first approximation, where owing to Remark 16, we take sin(*πx*) as the input function; that is, this function does not satisfy damping conditions on the plate's contour. A solution to the obtained ODEs was carried out via the difference method (FDM) with the plate partition 60 × 60 and successive solution to the obtained algebraic equations by the Gauss method.

Results of the quarter plate deflection function obtained on the line $\overset{-}{y}=0.5$ are given in Table 2. These results coincide with the conclusion of Theorem 12 regarding monotonous series {*a*_*k*_} behavior. The exact value of deflection equal to 0.0138 is taken from monograph [20].

## 5. Numerical Study of the Karman Equations 

### 5.1. Iterative Procedure of Linearization and Variational Iteration for System (6)

A simultaneous use of the iterative procedure is described in Section 3 and the method of variational iterations of system (6) allows us to carry out three remarkable procedures:

1. decrease of the order of the system twice (from the 8th to 4th order);
2. linearization of the sought nonlinear systems;
3. transition from PDEs to ODEs with constant coefficients.

This result is particularly important in the analysis of elliptic type PDEs.

Next, we present numerical results of our method using an example of the computation of flexible isotropic square plates of constant thickness for three types of boundary conditions (114)–(116).

Consider the following:

(114) $$\begin{array}{c} w=\frac{\partial^{2}w}{\partial n^{2}}=F=\frac{\partial^{2}F}{\partial n^{2}}=0,\;x=y=0,\;x=y=1, \end{array}$$

(115) $$\begin{array}{c} w=\frac{\partial^{2}w}{\partial n^{2}}=F=\frac{\partial F}{\partial n}=0;\;x=y=0;\;x=y=1, \end{array}$$

(116) $$\begin{array}{c} w=\frac{\partial w}{\partial n}=F=\frac{\partial F}{\partial n}=0;\;x=y=0;\;x=y=1. \end{array}$$

**Figure 2:**
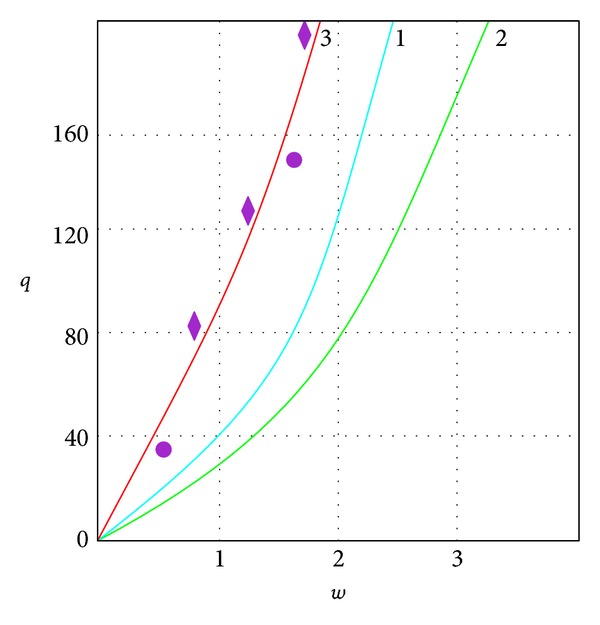
*q* versus *w*.

For simplicity, we apply the MVI using its first approximation *N* = 1. ODEs are reduced to AE (algebraic equations) through FDM with approximation 0(*h* ^2^), which is solved using the Gauss method. The interval of integration [0, 1] was divided into 100 parts. Relation *q*[*w*(0.5,0.5)] is illustrated in Figure 2. Curves (1), (2), and (3) refer to boundary conditions (114), (115), and (116), respectively. Curves (2) and (3) are obtained for the Poisson coefficient *ν* = 0.33 and curve (1) for *ν* = 0.1. Circles refer to experimental results [21]; stars refer to the solution obtained by FDM [22], where FDM was applied directly to (2) and nonlinear AEs was solved by Newton's method. Plane mesh step is 20 × 20. Computations were carried out with step Δ*q* = 10, where in order to accelerate convergence of the iterative procedure, *w* and *F* were taken from the previous step.

**Figure 3:**
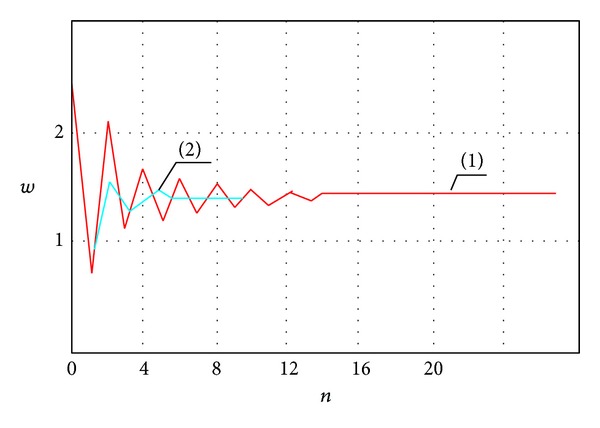
*w* versus *n*.

Dependence of the deflection change in the plate center on the number of iterations is shown in Figure 3 (curve (1)). Boundary conditions correspond to the plate support on flexible noncompressed ribs (114). The remaining parameters are *N* = 20, *q* = 60, *ν* = 0.28, and *ε* = 10^−3^. We require 16 iterations to achieve a priori given accuracy.

One may see from Figure 3 that the deflection oscillates in the vicinity of a certain averaged value, and it tends to it with an increase of the iteration number. It can be explained in the following way. Since the initial approximation is given by the linear equations, the observed deflection shall be larger than a real one. Substitution of *w* into the second equation of system (6) shows that the stress function value is also larger than a real one. Therefore, taking into account the obtained values of the stress function *F*, the deflection estimated through the first equation of system (6) is lesser than the real one. In other words, the deflection obtained via odd (even) iteration is lesser (larger) than the real one, and it can be estimated by the following formula:

(117) $$\begin{array}{c} w=\frac{w_{\text{odd}}+w_{\text{even}}}{2}. \end{array}$$

Formula (117) allowed us to reduce the number of iterations up to seven (see Figure 3, curve (2)). However, an increase of the load implies the convergence decrease.

### 5.2. Iterative Linearization Procedure (Poisson-Type Equations)

**Figure 4:**
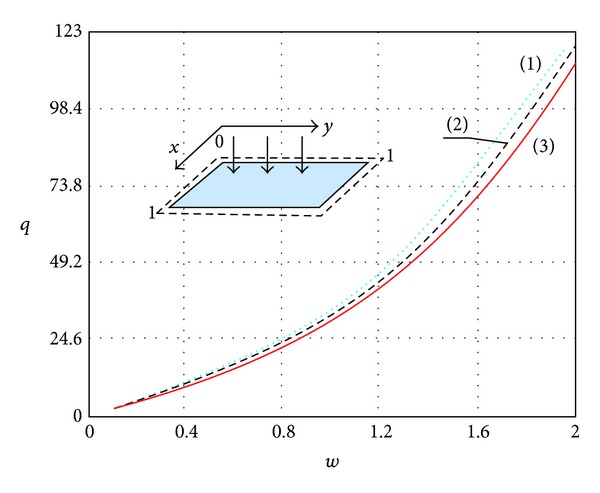
Load deflection function.

In order to solve system (89) we used the MVI and FDM. Results of a comparison of solutions for systems (6) and (89), using the MVI and FDM applied to system (2) in the case of a square plate, are shown in Figure 4. One may see that the result obtained by the application of procedure (6)—curve (2)—and (89)—curve (3)—differs slightly from the result obtained via the FDM—(1). Furthermore, a comparison of the results obtained through iterative procedures (6) (dot curve) and (89) (dashed curve) shows that the compared results for small deflection practically coincide.

**Figure 5:**
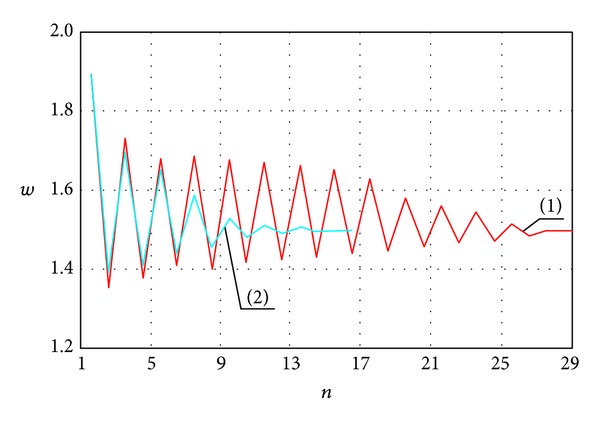
Plate deflection versus number of iterations.

The use of formula (117), in order to increase the convergence, implies that the function “load-deflection” practically remains unaffected, but the number of iterations increases.  Figure 5 shows results regarding the procedure (89): (i) without the application of (117)—curve (1)—and (ii) with the application of formula (117)—curve (2). One may observe that the iterative process converges twice as fast.

## 6. Summary

In this work we have proposed and theoretically established (theorems with proofs) some iterative procedures dedicated to a decrease of the order and then linearization of the Karman nonlinear PDEs. It has been shown that the result obtained via the modified Kantorovich-Vlasov method coincides with the exact solution. Furthermore, the theoretical considerations have been supported by the numerical analysis of the Karman equations using two introduced iterative procedures.

## Figures and Tables

**Table 1:** Solution methods.

Reduction to the Poisson-type equation (MVI)	Bubnov's method	Solution in series
0.004054	0.00416	0.00406

**Table 2:** Deflection of the plate quarter.

Step	$\overset{-}{y}=0.5,\;\;\overset{-}{x}=0.1$	$\overset{-}{y}=0.5,\;\;\overset{-}{x}=0.3$	$\overset{-}{y}=0.5,\;\overset{-}{x}=0.5$
1	0.55543336 · 10^−3^	0.19404364 · 10^−2^	0.22896260 · 10^−2^
2	0.16923198 · 10^−2^	0.79178517 · 10^−2^	0.10638649 · 10^−1^
3	0.21179814 · 10^−2^	0.10124784 · 10^−1^	0.13723563 · 10^−1^
4	0.21349905 · 10^−2^	0.102096 · 10^−1^	0.13840985 · 10^−1^
5	0.21349905 · 10^−2^	0.10211107 · 10^−1^	0.13842917 · 10^−1^
6	0.21352879 · 10^−2^	0.10211157 · 10^−1^	0.13843016 · 10^−1^
